# Supplementary material for: Microneedle Patterning of 3D Nonplanar Surfaces on Implantable Medical Devices Using Soft Lithography
Source: Micromachines (Basel). 2019 Oct 16;10(10):705. doi: 10.3390/mi10100705 (PMC6843263; doi:10.3390/mi10100705)
Supplement: Supplementary file 1 [file micromachines-10-00705-s001.pdf]

micromachines

Supplementary Materials

Microneedle Patterning of 3D Nonplanar Surfaces on Implantable Medical Devices Using Soft Lithography

Sun-Joo Jang [1,2], Tejas Doshi [1,2], Jerusalem Nerayo [2], Alexandre Caprio [1,2], Seyedhamidreza Alaie [1,2], Jordyn Auge [1,2], James K. Min [1,2], Bobak Mosadegh [1,2,*] and Simon Dunham [1,2,*]

1 Dalio Institute of Cardiovascular Imaging, NewYork-Presbyterian Hospital and Weill Cornell Medicine, New York, NY 10021, USA; drjalive@gmail.com (S.-J.J.); tnd2001@med.cornell.edu (T.D.); aac2009@med.cornell.edu (A.C.); sea2012@med.cornell.edu (S.A.); jordynauge@gmail.com (J.A.) jkm2001@med.cornell.edu (J.K.M.)

2 Department of Radiology, Weill Cornell Medicine, New York, NY 10021, USA; jdna2017@mymail.pomona.edu (J.N.)

* Correspondence: bom2008@med.cornell.edu (B.M.); sid2012@med.cornell.edu (S.D.); Tel.: +1-212-327-7170 (S.D.)

List:

Video S1: Lateral pull-out test for microneedle array under compression between slide glasses and porcine aorta.
Figure S1: Alternative microneedle templates from laser-cutting method.
Figure S2: Confocal microscopy of microneedles before and after pressurization.
Figure S3: Magnified view of microneedle array after pressurized stent implantation.
Figure S4: Lateral pull-out test for microneedle array.
Table S1: Comparison of different polymeric microneedle fabrication methods.

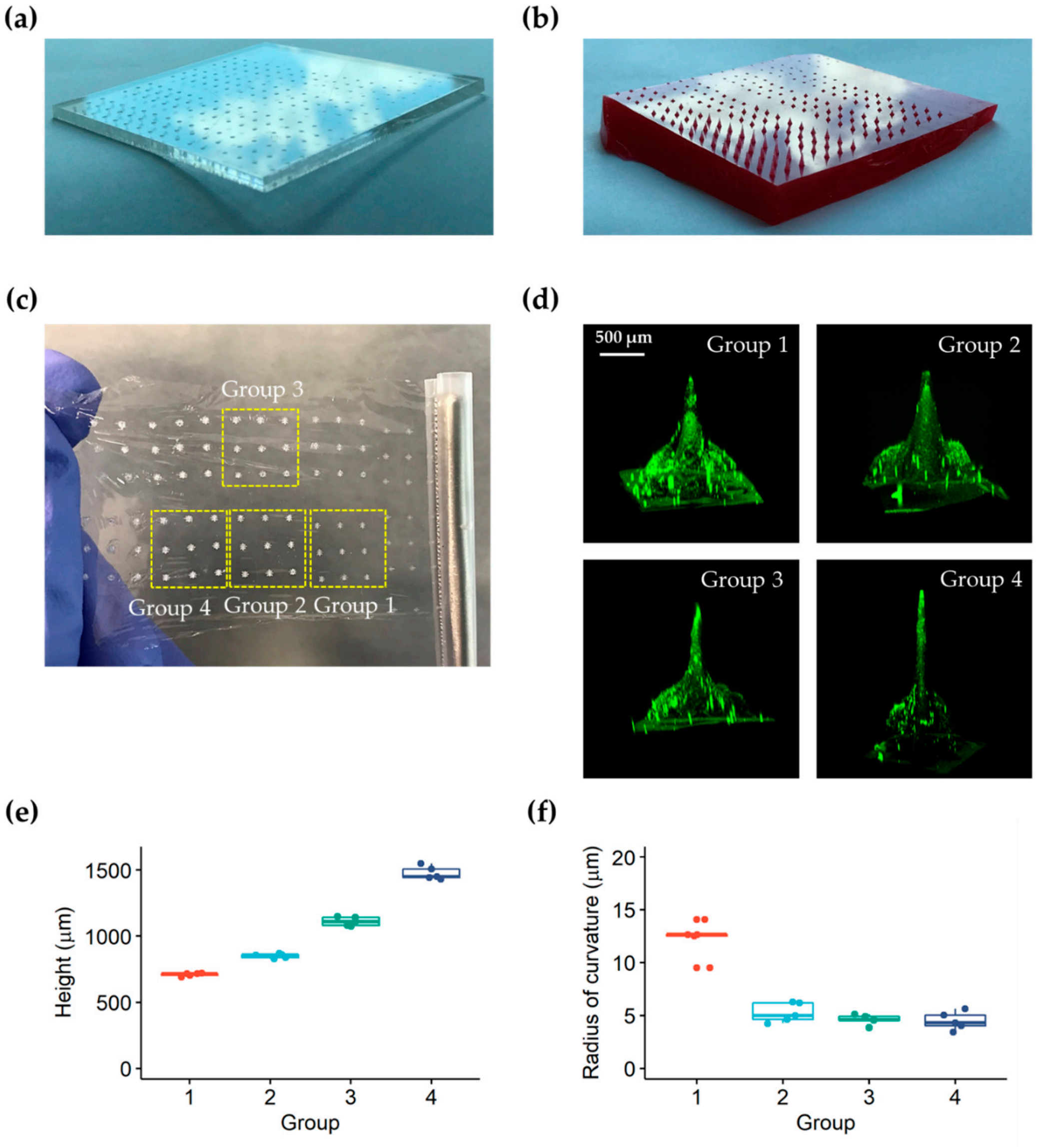

Figure S1. Alternative microneedle templates fabricated by a laser-cutting method. (**a**) Acrylic inverse template for various sizes of microneedles; (**b**) Sample of silicon microneedles generated using an inverse mold; (**c**) Thin microneedle film fabricated using the laser-cut method (four different groups for measurements are marked with dashed yellow boxes); (**d**) Volumetric image of four representative microneedles by confocal microscopy; (**e**) Distribution of height among the four microneedle groups ($P < 0.001$); (**f**) Distribution of radius of curvature among the four microneedle groups ($p < 0.001$).

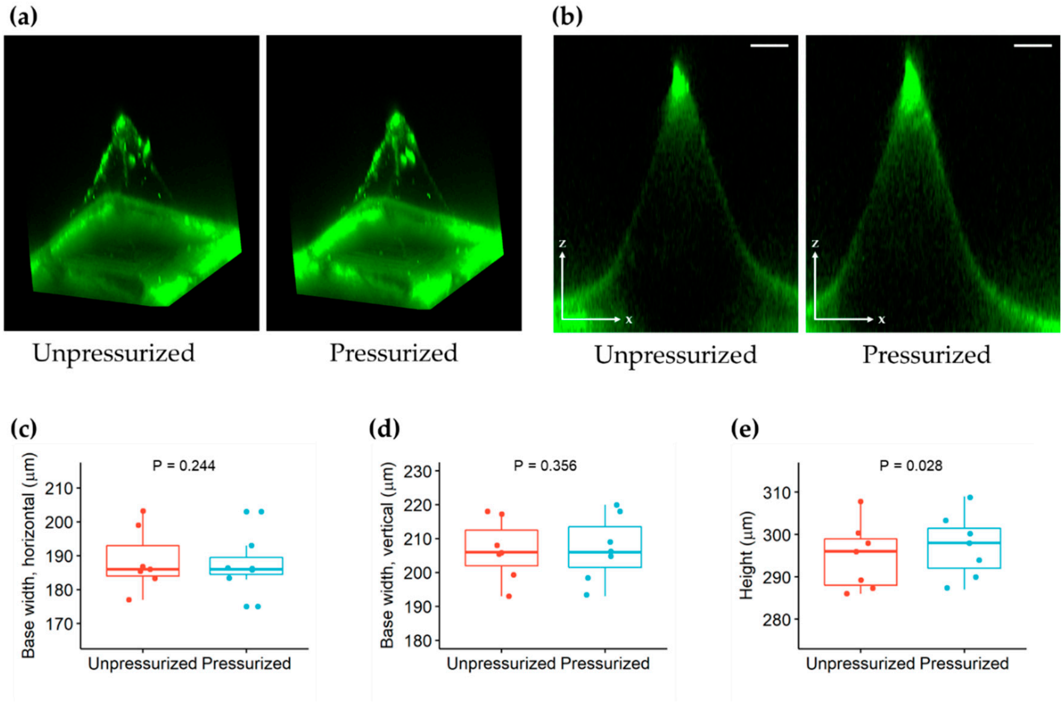

Figure S2. Confocal microscopy of microneedles before and after pressurization. (**a**) 3D maximum-intensity projection image of the microneedle before and after pressurization; (**b**) XZ-plane sectional view of the microneedle before and after pressurization; (**c**) Comparison of the horizontal base width of the microneedle before and after pressurization; (**d**) Comparison of the vertical base width of the microneedle before and after pressurization; (**e**) Comparison of the height of the microneedle before and after pressurization. Scale bar, 50 µm.

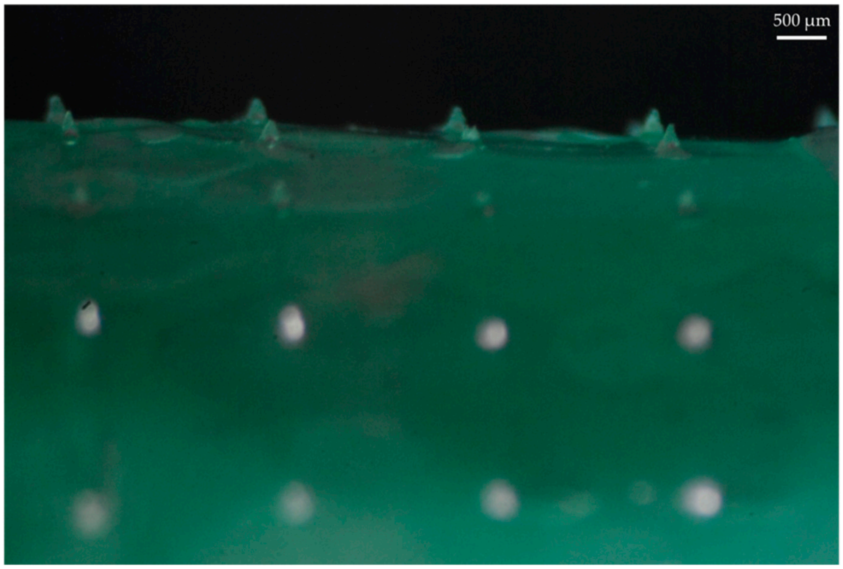

Figure S3. Magnified view of the microneedle array after pressurized stent implantation

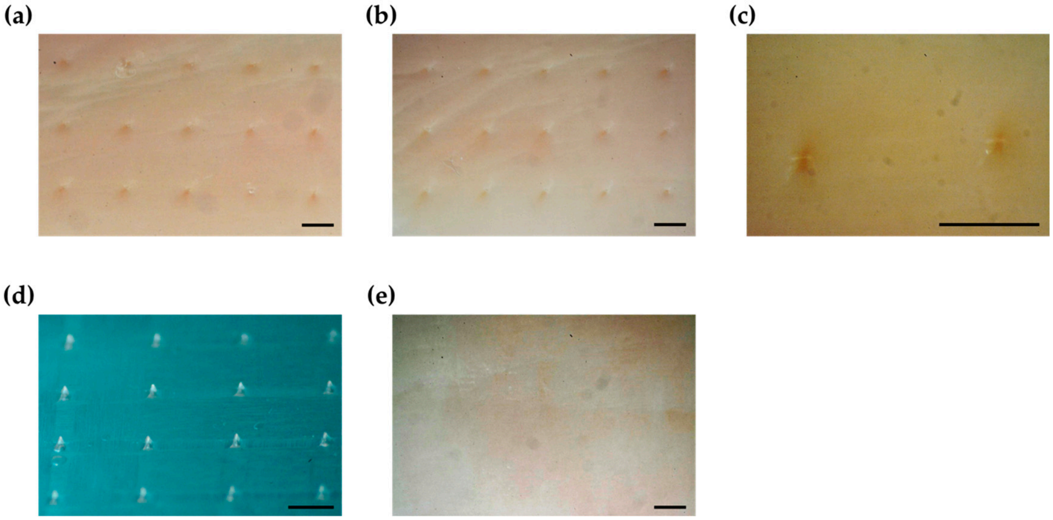

Figure S4. Lateral pull-out test on the microneedle array. (**a**) Porcine aorta and the microneedle array film were compressed together between slide glasses; (**b**) A lateral force was applied to the microneedle film and increased gradually. There was no mechanical failure of the microneedle; (**c**) Magnified view of the microneedles sliding over the porcine aorta; (**d**) Microneedle shape and sharpness were intact after the lateral pull-out experiment; (**e**) The porcine aorta was free from any damage or indent. Scale bar, 1 mm.

Table S1. Comparison of different polymeric microneedle fabrication methods.

Groups	Fabrication Techniques	Characteristics of MN	Substrate Material	Height of MN	Thickness of Film Layer	Potential Application
Pérennès et al.[1]	Deep X-ray lithography, electroplating, sactificial mold in PVA, PDMS micromold	Hollow MN	PMMA	500~700 μm	N/A	Transdermal drug delivery
Yung et al.[2]	Stainless steel microinjection molds, picosecond laser	Hollow MN	POM	500 μm	~200 μm	Transdermal drug delivery
Park et al[3]	Photolithography, micro-electromechanical masking and etching, PDMS mold, sacrificial polymer,	Biodegradable MN	PLA, PGA, PLGA	700~1500 μm	N/A	Transdermal drug delivery
Yang et al.[4]	Photolithography, PDMS mold	Swellable MN	PS, PS-*b*-PAA	700 μm	500~1000 μm	Adhesive on skin and intestine, drug delivery
Johnson et al.[5]	Stereolithography with CLIP technique	Sharp, tunable and biocompatible MN	TMPTA, PEG, PCL, PAA	400~1000 μm	1000 μm	Transdermal drug delivery

Nejad et al.[6]	CO_2 laser cutter, PDMS mold, casting	Low-cost scalable PDMS solid microneedles	PVA	1000~3000 μm	~500 μm	Transdermal drug delivery
Current technique	Soft lithography (PDMS mold, spin coating, casting)	Rigid microneedles on flexible film	TPU	300 μm	~50 μm	Enhanced anchorage for implantable medical devices

Abbreviations. MN, microneedle; PVA, polyvinyl acid; PDMS, polydimethylsiloxane; PMMA, polymethylmethacrylate; POM, polyoxymethylene; PLA, polylactic acid; PGA, polyglycolic acid; PLGA, poly(lactic-co-glycolic acid); PS, polystyrene; PS-b-PAA, polystyrene-*block*-poly(acrylic acid); CLIP, continuous liquid interface production; TMPTA, trimethylolpropane triacrylate; PEG, polyethylene glycol; PCL, polycaprolactone; PAA, polyacrylic acid; TPU, thermoplastic polyurethane; N/A, not available.

References

1. Perennes, F.; Marmiroli, B.; Matteucci, M.; Tormen, M.; Vaccari, L.; Di Fabrizio, E. Sharp beveled tip hollow microneedle arrays fabricated by LIGA and 3D soft lithography with polyvinyl alcohol. *J. Micromech. Microeng.* **2006**, *16*, 473.
2. Yung, K.L.; Xu, Y.; Kang, C.; Liu, H.; Tam, K.F.; Ko, S.M.; Kwan, F.Y.; Lee, T.M.H. Sharp tipped plastic hollow microneedle array by microinjection moulding. *J. Micromech. Microeng.* **2012**, *22*, 015016.
3. Park, J.H.; Allen, M.G.; Prausnitz, M.R. Biodegradable polymer microneedles: fabrication, mechanics and transdermal drug delivery. *J. Controlled Release* **2005**, *104*, 51–66.
4. Yang, S.Y.; O'Cearbhaill, E.D.; Sisk, G.C.; Park, K.M.; Cho, W.K.; Villiger, M.; Bouma, B.E.; Pomahac, B.; Karp, J.M. A bio-inspired swellable microneedle adhesive for mechanical interlocking with tissue. *Nat. Commun*. **2013**, *4*, 1702.
5. Johnson, A.R.; Caudill, C.L.; Tumbleston, J.R.; Bloomquist, C.J.; Moga, K.A.; Ermoshkin, A.; Shirvanyants, D.; Mecham, S.J.; Luft, J.C.; DeSimone, J.M. Single-Step Fabrication of Computationally Designed Microneedles by Continuous Liquid Interface Production. *PLoS One*. **2016**, *11*, e0162518.
6. Nejad, H.R.; Sadeqi, A.; Kiaee, G.; Sonkusale, S. Low-cost and cleanroom-free fabrication of microneedles. *Microsys. Nanoeng.* **2018**, *4*, 17073.

© 2019 by the authors. Submitted for possible open access publication under the terms and conditions of the Creative Commons Attribution (CC BY) license (http://creativecommons.org/licenses/by/4.0/).
